# Evolution of spliceosomal introns following endosymbiotic gene transfer

**DOI:** 10.1186/1471-2148-10-57

**Published:** 2010-02-23

**Authors:** Nahal Ahmadinejad, Tal Dagan, Nicole Gruenheit, William Martin, Toni Gabaldón

**Affiliations:** 1 Institut für Botanik III, Heinrich-Heine Universität Düsseldorf, Universitätsstr 1, 40225 Düsseldorf, Germany; 2 Max Planck Institute for Plant Breeding Research, Dept. Plant-Microbe Interactions, Carl-von-Linné-Weg 10, 50829 Köln. Germany; 3 Bioinformatics and Genomics Programme, Centre for Genomic Regulation (CRG), Dr Aiguader, 88 Barcelona 08003, Spain

## Abstract

**Background:**

Spliceosomal introns are an ancient, widespread hallmark of eukaryotic genomes. Despite much research, many questions regarding the origin and evolution of spliceosomal introns remain unsolved, partly due to the difficulty of inferring ancestral gene structures. We circumvent this problem by using genes originated by endosymbiotic gene transfer, in which an intron-less structure at the time of the transfer can be assumed.

**Results:**

By comparing the exon-intron structures of 64 mitochondrial-derived genes that were transferred to the nucleus at different evolutionary periods, we can trace the history of intron gains in different eukaryotic lineages. Our results show that the intron density of genes transferred relatively recently to the nuclear genome is similar to that of genes originated by more ancient transfers, indicating that gene structure can be rapidly shaped by intron gain after the integration of the gene into the genome and that this process is mainly determined by forces acting specifically on each lineage. We analyze 12 cases of mitochondrial-derived genes that have been transferred to the nucleus independently in more than one lineage.

**Conclusions:**

Remarkably, the proportion of shared intron positions that were gained independently in homologous genes is similar to that proportion observed in genes that were transferred prior to the speciation event and whose shared intron positions might be due to vertical inheritance. A particular case of parallel intron gain in the *nad7 *gene is discussed in more detail.

## Background

Many eukaryotic genes contain spliceosomal introns [1]: segments of non-coding sequences that are excised from the pre-mRNA by the spliceosome complex [2]. Spliceosomal introns have been found with huge varying rates in all sequenced eukaryotes and are absent in all prokaryotic genomes sequenced to date [3]. These findings have been discussed in the context of two alternative hypotheses. The introns-early hypothesis states that spliceosomal introns were present in the last common ancestor of prokaryotes and eukaryotes but were subsequently lost in all prokaryotes [4]. In contrast, the introns-late hypothesis links the origin of spliceosomal introns to the emergence of eukaryotes. In accordance to the introns-late hypothesis, spliceosomal introns were supposed to originate from self-splicing group II introns during the evolution of eukaryotes [5]. This model is supported by similarities between group II introns and the catalytic snRNA components of the spliceosome, suggesting that they might have had a common ancestor [6,7]. The fact that group II introns are found in bacterial and mitochondrial genomes suggests a possible evolutionary connection between spliceosomal introns and the development of mitochondria [8,9]. These cell organelles originated by endosymbiosis from an alpha-proteobacterial ancestor [10]. In the course of evolution, their genomes were reduced through gene loss but also to a large extent through the transfer of many genes to their host genome [11,12]. These endosymbiotic gene transfers could have spread group II introns into the host genome, which, in turn, might have initiated the evolution of spliceosomal introns and the spliceosome. Additionally, these influences might have also resulted in a selective force towards the evolution of a nucleus which forms physical boundaries between the splicing and translation processes [9].

The introns-early and introns-late hypotheses have been discussed in the literature until recently [13,14] with every new sequenced genome adding more information to our understanding of intron evolutionary dynamics throughout eukaryotic lineages. Nowadays there is a larger consensus around the introns-late hypothesis, although the mechanisms and dynamics of intron gains and loss in eukaryotes are still a matter of debate. Recently, many studies have focused on inferring rates of intron gain and loss across the evolution of eukaryotes. The results reveal large differences in intron gain and loss rates in different lineages [15-21]. Other studies have traced the evolution of introns across the major eukaryotic lineages by using different evolutionary models [22,23].

One of the difficulties of modeling intron-evolution is that ancestral gene structures are generally unknown. Therefore, models rely on certain parameters that are used to infer ancestral states of intron presence or absence. We circumvent this problem here by using a set of nuclear genes that originated by endosymbiotic gene transfer. These genes did not contain spliceosomal introns when they were transferred to the host genome, so that the introns found in these genes must all have been gained after the integration of the gene. We exploit the circumstance that nuclear encoded genes with mitochondrial origin can be identified by their sequence similarity and phylogenetic proximity to their alpha-proteobacterial homologs [11,24]. In particular, we put our focus on genes with a clear-cut proto-mitochondrial origin, as reported by phylogenetic analyses of mitochondrial ribosomal proteins [25] and protein complexes from the oxidative phosphorylation pathway (OXPHOS) [11,26,27]. Our results reveal a highly dynamic species-specific intron evolution, which is able to shape relatively rapidly the intron-exon structure of a transferred gene. Hence, intron density, exon symmetry and intron phase distribution of recently transferred genes is similar to other genes in the genome. We find several instances of independent parallel transfers of genes. Comparing their ratio of shared intron positions to those of genes that vertically derive from a single transfer event, our results indicate that, for our set of genes, the proportion of shared intron positions between genes that were transferred independently on more than one occasion is similar to those that were transferred in a single event. Finally, we provide an in-depth analysis of clear-cut case of an intron that was inserted at identical positions in the *nad7 *gene which was transferred twice independently in the plant and animal lineages.

## Results and discussion

### Proto-mitochondrial derived genes are not different from other genes in terms of their intron structure

**Figure 1:**
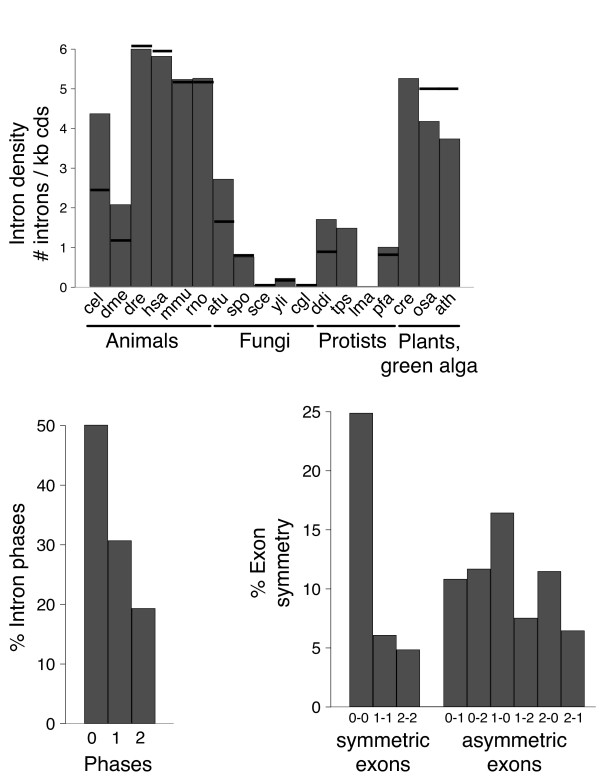
**Intron densities and distributions of intron phases and exon symmetry are shown for all proto-mitochondrial genes of the oxidative phosphorylation pathway and the ribosomal mitochondrial proteins and their homologs**. The intron density is given as the number of introns per 1 kb of coding sequence for each species for the groups animals (cel: *Caenorhabditis elegans*, dme: *Drosophila melanogaster*, dre: *Danio rerio*, hsa: *Homo sapiens*, rno: *Rattus norvegicus*), fungi (afu: *Aspergillus fumigatus*, spo: *Schizosaccharomyces pombe*, sce: *Saccharomyces cerevisiae*, yli: *Yarrowia lipolytica*, cgl: *Candida glabrata*), protists (ddi: *Dictyostelium discoideum*, tps: *Thalassiosira pseudonana*, lma: *Leishmania major*, pfa: *Plasmodium falciparum*), and plants/green alga (cre: *Chlamydomonas reinhardtii*, osa: *Oryza sativa*, ath: *Arabidopsis thaliana*). The average intron densities for the different species are indicated by horizontal lines, values were taken or computed from the literature [38,57-59]. Intron phases are presented in percentages for all genes. The percentages of exon symmetry are shown separately for symmetric and asymmetric exons, in which all possible symmetries are considered.

We compiled a list of 64 nuclear-encoded human genes of proto-mitochondrial origin [11,25-27]. These include 44 genes that encode for proteins of the mitochondrial ribosome and 20 genes of the oxidative phosphorylation (OXPHOS) pathway (Additional file 1). The intron-exon structure of these genes and their homologs across a broad set of 18 eukaryotic organisms was determined by comparing each protein sequence with the respective genomic sequence (see Methods). The set of eukaryotic genomes includes three plant/green alga genomes, five fungi, six metazoans and four protists (Additional file 2). The distribution of intron densities, intron phases, and symmetric and asymmetric exons in proto-mitochondrial derived OXPHOS and ribosomal genes are shown in Figure 1. Intron densities range from 0 to 6 introns/kb of coding sequence and always show ranges that are within the normal values of the species considered [14]. The same can be observed for other characteristics such as the prevalence of phase 0 introns and symmetrical exons. A bias of phase 0 introns is a frequent observation, which is often linked with the preference of newly gained introns [28,29]. A ratio of 5:3:2 of phase 0, phase 1 and phase 2 introns as found in this study for the considered proto-mitochondrial genes is in accordance with results reported for genes of different origins [30,31]. Finally, our finding that 0-0 exons account for the majority of symmetrical exons is also in line with general observations in eukaryotic genomes [30]. Thus, according to their intron densities, exon symmetries and phase distributions, proto-mitochondrial derived OXPHOS and ribosomal genes are undistinguishable from other genes in eukaryotic genomes. In a similar study with chloroplast-derived genes in plant genomes, Basu et. al. [32], found significant, but only slightly lower intron densities in those genes transferred from the chloroplast than in ancestral eukaryotic genes. In contrast, in a study by Roy et al. [33] little intron gain was detected in genes acquired by lateral transfer from prokaryotic donors.

### Lack of correlation between time of endosymbiotic gene transfer and intron density

**Figure 2:**
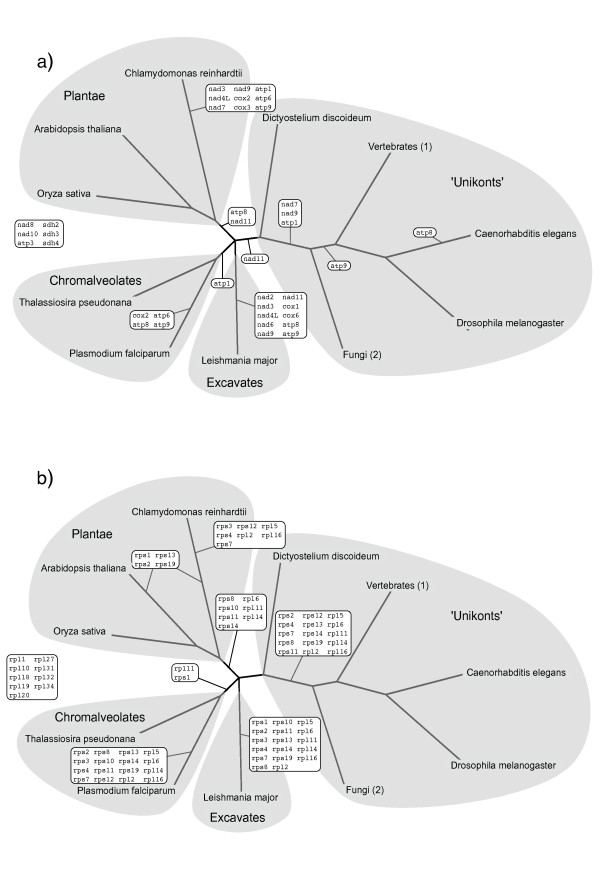
**The tree represents current view of phylogenetic relationships between the lineages sampled in this analysis, as summarized by Roger and Simpson **[**60**]. Inferred timing for the transfers of genes from the mitochondrion to the host nucleus is labeled at the branches. The timing of each transfer depends on the presence or absence of each gene in the mitochondrial genome and the phylogeny. Proto-mitochondrial genes of a) the oxidative phosphorylation pathway, b) ribosomal mitochondrial proteins.

**Figure 3:**
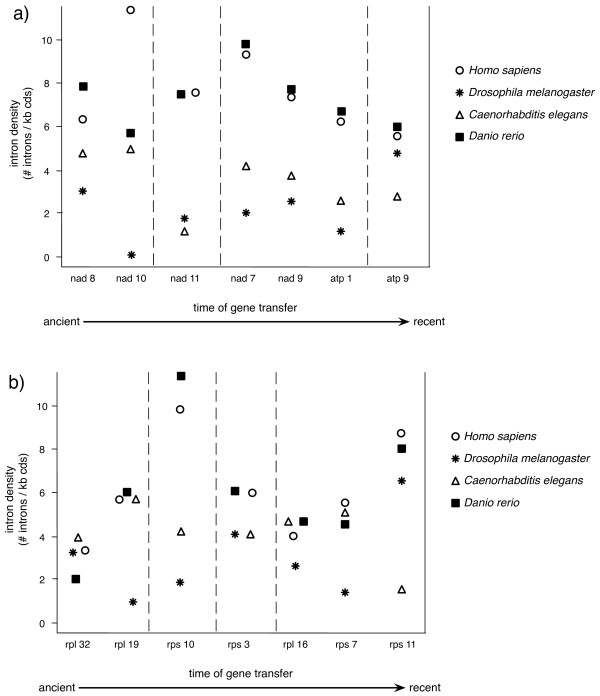
**The intron density is shown for genes that were transferred at different time scales during evolution from the mitochondrion to the nucleus in *Homo sapiens, Drosophila melanogaster, Caenorhabditis elegans *and *Danio rerio***. a) proteins of the oxidative phosphorylation pathway, b) ribosomal mitochondrial proteins. Although the most ancient class of transfers (nad8, nad10, rpl32, rpl19) is unassigned in Figure 2 we consider them to be relatively more ancient than nad11, rps10 and rps3 because the latter are nuclear only in unikonts and the former are nuclear in most eukaryotic groups.

Mapping the relative time of endosymbiotic gene transfer from the mitochondrion (see Methods) onto the phylogenetic tree of eukaryotes [34], and considering a parsimonious scenario, we can approximate the history of endosymbiotic gene transfers to the nuclear genome, and thereby establish a relative ordered timing of the events (Figure 2). It must be noted, that a parsimonious approach might be affected by incomplete taxonomic sampling and errors in the species tree. To limit such effects we used all available data on mitochondrial genomes available at NCBI database and left unresolved those transfers that could not be placed with confidence due to multifurcations in the tree of eukaryotes. This approach served to establish a relative timing of endosymbiotic gene transfer events for some genes and taxonomic groups that are in resolved parts of the tree for which mitochondrial genomes are well sampled. In particular, for genes transferred within the metazoan lineage, which is densely sampled in terms of mitochondrial genomes, we could classify genes into relatively more ancient and more recent transfers. In order to test the variation of intron gain over time, we compared the intron densities of early and late transfers in genomes of four metazoan species: the vertebrates *Homo sapiens *and *Danio rerio*, the insect *Drosophila melanogaster *and the nematode *Caenorhabditis elegans *(Figure 3). Our results show no correlation between intron density and the time of the gene transfer. Instead, differences between the densities of the corresponding genes in different species are generally larger than the differences observed between genes transferred at different evolutionary stages. This indicates that intron densities are governed by lineage-specific constraints and are independent of the time of the transfer event. This is consistent with previous findings. For instance, an extensive lineage-specific loss of introns in an intron-rich ancestor is suggested to happened in some chromalveolate lineages [35]. Our results suggest that intron gain and not just reduced intron loss could be responsible for the current high densities found in plant and animal genomes. In fact, intron gain is the only process that can explain the current high intron densities in recently transferred genes. Nevertheless, the existence of an intron-rich ancestor of eukaryotes is strongly supported by a high rate of shared intron positions between animals, fungi and plants [22,23,36].

### Significant inter-kingdom conservation of plant-animal intron positions

To assess the extent of shared intron positions we aligned the protein sequences of transferred genes in the different lineages included in the study (see Methods). Consistent with previous results [36], most intron positions are shared between the most divergent groups animals and plants (Tables 1 and 2). The distribution of the number of species-specific introns reflects the overall intron density in each species. Comparing only those intron positions in highly-conserved alignment regions identified with Block Maker [37], the numbers of shared intron positions are reduced but still show the same trend (Tables 1 and 2). Only few introns are found at the same position across more than two groups of organisms. Three intron positions are shared between animals, plants and fungi. Also three introns at the same positions are shared between animals, fungi and *Dictyostelium discoideum*. Two shared intron positions are found in animals, plants and *Dictyostelium discoideum*.

**Table 1:** Number of species-specific and shared intron positions in proteins of the oxidative phosphorylation pathway.

	Animals^1^	Plants^2^	Fungi^3^	*Dictyostelium discoideum*	*Leishmania major*	*Plasmodium falciparum*	*Thalassiosira pseudonana*
Animals^1^	**287**	60 (8.62)	12 (1.72)	4 (0.58)	-	-	1 (0.14)
Plants^2^	4 (0.58)	**285**	1 (0.14)	-	-	-	3 (0.43)
Fungi^3^	1 (0.14)	-	**78**	-	-	-	-
*Dictyostelium discoideum*	1 (0.14)	-	-	**15**	-	1 (0.14)	-
*Leishmania major*	-	-	-	-	-	-	-
*Plasmodium falciparum*	-	-	-	-	-	**7**	-
*Thalassiosira pseudonana*	-	-	-	-	-	-	**24**

Species specific intron positions are shown in the diagonal. Shared intron positions between the different groups of species within the complete multiple protein alignments are shown above the diagonal, shared intron positions only within conserved regions of the alignments are shown below the diagonal. Percentage of shared positions is indicated in brackets. ^1^*Homo sapiens, Mus musculus, Rattus norvegicus, Danio rerio, Caenorhabditis elegans, Drosophila melanogaster*, ^2^*Arabidopsis thaliana, Chlamydomonas reinhardtii, Oryza sativa*, ^3^*Aspergillus fumigatus, Schizosaccharomyces pombe, Saccharomyces cerevisiae, Candida glabrata, Yarrowia lipolytica*.

**Table 2:** Number of species-specific and shared intron positions in Ribosomal mitochondrial proteins.

	Animals^1^	Plants^2^	Fungi^3^	*Dictyostelium discoideum*	*Leishmania major*	*Plasmodium falciparum*	*Thalassiosira pseudonana*
Animals^1^	**318**	12 (2.49)	4 (0.83)	3 (0.62)	-	-	2 (0.42)
Plants^2^	6 (1.25)	**105**	1 (0.21)	-	-	-	-
Fungi^3^	1 (0.21)	1 (0.21)	**17**	-	-	-	-
*Dictyostelium discoideum*	1 (0.21)	-	-	**6**	-	-	-
*Leishmania major*	-	-	-	-	-	-	-
*Plasmodium falciparum*	-	-	-	-	-	**11**	-
*Thalassiosira pseudonana*	1 (0.21)	-	-	-	-	-	**3**

See legend of table 1 for indications

The timing of the transfer events reveals independent transfers in different species, mostly involving the green alga *Chlamydomonas reinhardtii *and other groups (Figure 2). For instance, the genes *nad7, nad9 *and *atp1 *of the oxidative phosphorylation, were transferred twice independently in animals, fungi and in *Chlamydomonas reinhardtii *(Figure 2a). The same observation is made within the timing of gene transfer events of the mitochondrial ribosomal proteins (Figure 2b). Five gene transfers took place independently in *Chlamydomonas reinhardtii, Leishmania major, Plasmodium falciparum*, and before the split of animals and fungi (*rpl2, rpl5, rpl16, rps4, rps7*). A list of putative independent transfers is provided in Table 3.

**Table 3:** Genes that are independently transferred and which could be identified with their mitochondrial gene names.

Gene	Independent Transfers				
*nad7*	*C. reinhardtii*		Animals, Fungi		
nad9	*C. reinhardtii*		Animals, Fungi		
nad11	Plants and green alga		Animals, Fungi	*L. major*	*D. discoideum*
*rps2*	*C. reinhardtii*	*A. thaliana*	Animals, Fungi	*L. major*	*P. falciparum*
*rps11*	*C. reinhardtii*	*A. thaliana*	Animals, Fungi	*L. major*	*P. falciparum*
*rps14*	*C. reinhardtii*	*A. thaliana*	Animals, Fungi	*L. major*	*P. falciparum*
*rpl2*	*C. reinhardtii*		Animals, Fungi	*L. major*	*P. falciparum*
*rpl16*	*C. reinhardtii*		Animals, Fungi	*L. major*	*P. falciparum*
*rpl7*	*C. reinhardtii*		Animals, Fungi	*L. major*	*P. falciparum*
*rpl12*	*C. reinhardtii*		Animals, Fungi		*P. falciparum*
*rpl11*	Plants and green alga		Animals, Fungi	*L. major*	*P. falciparum* *T. pseudonana*
*rpl14*	Plants and green alga		Animals, Fungi	*L. major*	*P. falciparum*

The first three genes (*nad*x) are genes of the oxidative phosphorylation pathway, the other nine genes (*rps*x/*rpl*x) are genes of ribosomal mitochondrial proteins of the small and the large ribosomal subunit, respectively.

The large number of independently transferred genes in the green algal lineage allows us to compare the occurrence of shared intron positions between genes transferred independently and those derived from a common nuclear-encoded ancestor. The observation that most shared intron positions are found between distantly-related species can be explained either by conservation of intron positions from a common ancestor or by parallel intron gain. Different evolutionary models infer different rates of parallel intron insertion. For Qiu and colleagues most shared intron positions should be gained independently [31], whereas most other models provide lower estimates (5-18%) for the fraction of shared intron positions that result from independent insertions [23,38,39].

Shared positions between distant species such as animals and plants have been considered ancient positions [22,23], considering that the probability for an independent gain of two introns at the same position is very small. Our data, however, show that this is not necessarily the case. In both, the proto-mitochondrial genes *nad7 *and *nad11 *which were independently transferred in the eukaryotes under consideration (Table 3) and the gene *sdh2 *which was transferred in the basal eukaryotic lineage (Figure 2a; transfer at the root of the tree) shared intron positions were identified between the green alga *Chlamydomonas reinhardtii *and some of the animals (*Homo sapiens, Mus musculus, Rattus norvegicus*). A comparison of the percentage of those shared intron positions between these groups reveals almost a double amount of positions in the genes that were transferred independently (4.38%) in contrast to shared positions in the other genes (2.52%). This means that at large evolutionary distances shared positions are not always indicative of the prevalence of ancestral intron positions. The percentage of shared positions between *Chlamydomonas *and animals in these genes is remarkably lower than previous reports that set a ~23% of shared introns between human and *Arabidopsis *genes [36]. However, it must be noted that the specific nature and reduced size of our dataset makes it difficult to extrapolate our findings to a general case. For the gene *nad7*, the phylogenetic distribution in nuclear and mitochondrial genomes and its evolutionary history which includes a parallel intron gain was reconstructed in detail.

### An unambiguous parallel intron gain at identical sites in two independently transferred nad7 genes

**Figure 4:**
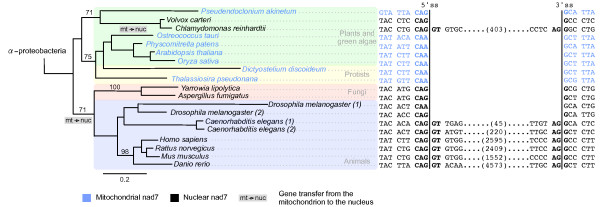
**A comprehensive phylogeny of the gene *nad7 *including mitochondrial and nuclear encoded homologs**. The tree is rooted by alpha-proteobacterial homologs. The two independent gene transfers of *nad7 *from the mitochondrion to the nucleus are labelled at the tree. The nucleotide region of the shared intron position is shown. The different lengths of the intron sequences are indicated in parentheses, the splicing sites are marked in bold

To gain a more detailed insight into the parallel insertion of introns at identical positions we present here in detail a particular example from our dataset, that of a parallel intron in the *nad7 *gene. The gene *nad7 *was transferred independently before the split of animals and fungi and in the green alga *Chlamydomonas reinhardtii *and the only shared intron position was found between animals and the green alga. To gain a more detailed view of the evolution of the gene *nad7*, we added to the phylogenetic analysis also the mitochondrial encoded homologs of the two protists *Dictyostelium discoideum, Thalassiosira pseudonana*, the plants *Arabidopsis thaliana, Oryza sativa*, the green algae *Pseudendoclonium akinetum, Ostreococcus tauri *and the moss *Physcomitrella patens*, as well as the nuclear encoded *nad7 *gene of the green alga *Volvox carteri*. The presence of the gene *nad7 *in the mitochondrial genome in all other plants, the moss and the two green algae *Pseudendoclonium akinetum *[40] and *Ostreococcus tauri *[41] supports the evolutionary scenario of independent transfer in the two green algae *Chlamydomonas reinhardtii, Volvox carteri*, and the animal/fungi split. These at least two independent transfer events are also supported by the reconstructed phylogenetic tree that contains both, nuclear and mitochondrial genes as well as alpha-proteobacterial *nad7 *homologs as the outgroup (Figure 4).

The nuclear encoded *Chlamydomonas reinhardtii nad7 *gene possesses 11 introns. A single shared intron position is found in a conserved region of the alignment between the green alga *Chlamydomonas reinhardtii *and the animals. The introns are all of phase 0 at exactly the same position of the gene as shown in Figure 4. In all sequences, the codon before the intron position codes for the amino acid glutamine. With one exception each, two different codons are used in the nuclear and the mitochondrial encoded genes for glutamine. There is a CAA found in the mitochondrial genes and a CAG in the nuclear genes, in agreement with a different average codon usage in mitochondrial and nuclear genes (Additional file 3). The codon before the intron together with the first nucleotide G after the intron correspond to a classical proto-splice site (C|A)AG - (A|G) (Figure 4) [42].

Interestingly, the shared intron position is surrounded by two group II introns in the mitochondrial sequences of the moss *Physcomitrella patens *and the plants *Arabidopsis thaliana *and *Oryza sativa*, 15 codons upstream and eight codons downstream, respectively (Additional file 4). Although it might be tempting to speculate on a possible role of these surrounding group-II introns in the formation of the spliceosomal intron after the transfer, the fact that such introns are rare, if not completely absent in most mitochondrial genomes, implies that most introns in recently transferred genes have been formed by alternative mechanisms. Altogether, our observations indicate that the gene *nad7 *was transferred twice independently and subsequently adapted its codon usage to that of nuclear genes. This originated the presence of a proto-splice site in the sequence of the *nad7 *gene, which, in turn, enabled the insertion of an intron at the same position in the different lineages.

## Conclusions

Arguments in favor of intron antiquity at identical intron positions are generally founded in weighing the relative probabilities of massive intron loss versus a few parallel intron gains [14,23]. Although several clear-cut cases of parallel intron gains have been previously described [43], this process is still considered a rarity. Our results present several independent intron gains in homologous genes that were transferred independently from the mitochondrion to the nucleus, showing that independent acquisition of introns have been relatively frequent in this group of genes. In fact, for the cases we have examined in more detail, the number of parallel intron gains is similar to the fraction of conserved shared positions at the same evolutionary distance. These results, albeit based on a limited sample of a specific set of genes, indicate that shared intron positions can, in some instances, arise independently by parallel insertions in distantly-related lineages.

## Methods

### Sequence data

All human nuclear encoded genes of the oxidative phosphorylation pathway and mitochondrial ribosomal proteins were obtained from the SwissProt database [44]. Genomic nucleotide and protein sequences of 18 completely sequenced eukaryotes were downloaded from GenBank [45] and JGI http://www.jgi.doe.gov/ databases as of March 2007. For both the nucleotide and the protein sequences, local databases were created. Three plant/green alga genomes were included in the analyses, *Arabidopsis thaliana, Oryza sativa*, and *Chlamydomonas reinhardtii*. Five fungal genomes, *Aspergillus fumigatus, Candida glabrata, Saccharomyces cerevisiae, Schizosaccharomyces pombe*, and *Yarrowia lipolytica *and six animal genomes, *Danio rerio, Drosophila melanogster, Caenorhabditis elegans, Homo sapiens, Mus musculus*, and *Rattus norvegicus*. Four different protist genomes were chosen, *Thalassiosira pseudonana, Plasmodium falciparum, Leishmania major*, and *Dictyostelium discoideum *(Additional file 2).

### Proto-mitochondrial genes

The information about the proto-mitochondrial origin of the mitochondrial ribosomal proteins was taken from [25]. The proteins of the oxidative phosphorylation pathway were downloaded from SwissProt [44] based on the information for the proteins of complex I [27]. To assign the corresponding mitochondrial gene names and to test again the proto-mitochondrial origin of the human genes of the oxidative phosphorylation pathway a BLAST [46] search was performed against the genome of the alpha-proteobacteria *Rickettsia prowazekii*. If the search resulted in a significant hit, annotated with a function in the electron transport chain, a second BLAST was performed against the mitochondrial genome of the protozoon *Reclinomonas americana *which has the largest number of mitochondrial-encoded proteins [47]. With the information about an existing homolog in *Reclinomonas americana*, the mitochondrial gene name could be assigned in some cases to the human nuclear encoded mitochondrial proteins.

To find eukaryotic homologs of the proto-mitochondrial genes, we used BLAST with each human protein as query and the protein database consisting of the 18 species. Resulting hits with an e-value < = 1e-06 were considered. For each set of homologs, a multiple protein sequence alignment was reconstructed using MUSCLE [48].

### Timing of endosymbiotic gene transfer events

The presence of genes in the mitochondrial genomes of the 18 species used in this study and other species was checked with the mitochondrial gene content tables in NCBI http://ncbi.nlm.nih.gov. The phylogenetic relationship between the 18 species [34] was used to assign the relative time of gene transfers regarding to speciation events. The relative timing of endosymbiotic gene transfer events were specified for both sets of proteins, the oxidative phosphorylation and the mitochondrial ribosomal proteins. Combining gene presence/absence information with the taxonomic relationships of the species results in a reconstruction of gene transfer events from the mitochondrion to the nucleus. Due to the uncertainty in some nodes of the eukaryotic tree and a sparse presence pattern of some genes in mitochondrial genomes, the timing for several transfer events were considered unresolved.

### Identification of intron positions

In the first step, BLAT [49] was used to align the protein sequence to the genomic sequence of the corresponding species. The result of BLAT is an alignment of the protein sequence to the exonic regions in the genome sequence without overlapping ends, where putative introns are not aligned. To identify the intron positions the following filtering steps were implemented in Perl scripts. The putative intron region had to be longer than 20 nucleotides and consists of a canonical splicing site, which means that nucleotides GT and AG are found at the beginning and the end of the sequence, respectively. To verify this inference, 18 nucleotides of the genomic region surrounding the putative splicing site were translated into protein sequence and compared with the query protein. If the translation was identical to the query sequence, the intron positions were identified together with the phase of each intron. A similar method for intron identification was recently published [50].

### Comparing intron positions

Presence/absence matrices of introns were built for each alignment to compare their positions. Shared intron positions are defined as introns that are found at exactly the same amino acid within the multiple protein sequence alignment. In addition, we determined shared intron positions only in conserved regions of the alignments. Therefore, conserved regions in each alignment were determined with Block Maker [37], a feature at Blocks database [51]. The intron density of a gene is given as the number of introns per 1 kb of coding sequence.

### Phylogenetic reconstruction of the nad7 gene

Protein sequences were aligned with MUSCLE [48], and all gapped sites were removed. Because the *nad7 *data sample includes eukaryotic nuclear sequences, mitochondrial sequences, and prokaryotic sequences, the phylogenetic reconstruction method has to take into account different evolutionary rates [52]. Therefore the ProtTest [53] program was used to estimate which substitution model fits the data best. The program computes maximum likelihood trees using phyml [54] under different substitution models and outputs the most likely tree according to different criteria. The maximum likelihood values for the trees are then used to perform a goodness of fit test with the AICc (Akaike Information Criterion with a second order correction for small sample sizes [55] and the BIC (Bayesian Information Criterion). In all cases the WAG [56] substitution model with an estimated proportion of invariable sites and a Γ-distribution (WAG+I+G) was chosen to explain the evolution of *nad7 *best. Bootstrap values were calculated using this model with 100 bootstrap replicates. The phylogenetic tree is rooted by the clade of alpha-proteobacterial *nad7 *genes.

## Authors' contributions

NA contributed to acquisition, analysis and interpretation of data and drafting the manuscript. TD contributed to conception and design of the work, analysis and interpretation of data and drafting the manuscript. NG contributed to acquisition, analysis and interpretation of data. WM contributed to conception and design of the work, interpretation of data and critical revision of the manuscript. TG contributed to conception and design of the work, interpretation of data and drafting and critical revision of the manuscript. All authors read and approved the final version of the manuscript.

## Supplementary Material

Additional file 1 Table of all human proto-mitochondrial genes of the a) oxidative phosphorylation pathway and the b) mitochondrial ribosome with their SwissProt ID and the corresponding mitochondrial gene name if it could be identified by BLAST against the mitochondrial genome of *Reclinomonas americana*. Click here for file

Additional file 2 Database sources of the complete genome and protein sequences. Click here for file

Additional file 3 Percentage of codon usage for the amino acid glutamine in a) the mitochondrial genomes and in b) the nuclear genomes of the species that are included in the analysis of the parallel intron gain in the gene *nad7*. Click here for file

Additional file 4 Part of the multiple protein alignment of the gene *nad7*. Click here for file
