# Supplementary material for: Evolution of spliceosomal introns following endosymbiotic gene transfer
Source: BMC Evol Biol. 2010 Feb 23;10:57. doi: 10.1186/1471-2148-10-57 (PMC2834692; doi:10.1186/1471-2148-10-57)
Supplement: Additional file 1 — Table of all human proto-mitochondrial genes of the a) oxidative phosphorylation pathway and the b) mitochondrial ribosome with their SwissProt ID and the corresponding mitochondrial gene name if it could be identified by BLAST against the mitochondrial genome of Reclinomonas americana. [file 1471-2148-10-57-S1.PDF]

Additional file 1: Table of all human proto-mitochondrial genes of the a) oxidative phosphorylation pathway and the b) mitochondrial ribosome with their SwissProt ID and the corresponding mitochondrial gene name if it could be identified by BLAST against the mitochondrial genome of *Reclinomonas americana*.

a)

SwissProt ID	mitochondrial gene name	SwissProt ID	mitochondrial gene name
Complex I		Complex III	
O75306	*nad7*	P08574	
O00217	*nad8*	P47985	
O75489	*nad9*	Complex IV	
O75251	*nad10*	P99999	
P28331	*nad11*	Complex V	
P19404		P06576	*atp1*
P49812		P25705	*atp1*
Complex II		P36542	*atp3*
P21912	*sdh1*	P48201	*atp9*
P31040	*sdh2*	Q06055	*atp9*
Q99643	*sdh3*	P05496	*atp9*
		P48047	

b)

SwissProt ID	mitochondrial gene name	SwissProt ID	mitochondrial gene name
Large ribosomal subunit		Q6P1L8	*rpl14*
O75394		Q5T653	*rpl2*
Q13084		Q9BYC8	*rpl32*
Q7Z2W9		Q9BYD2	
Q8TCC3		Q9HD33	
Q9BQ48	*rpl34*	Q9NWU5	
Q9BYC9	*rpl20*	Q9P015	
Q9BYD3		Q9Y3B7	*rpl11*
Q9H0U6	*rpl18*	Small ribosomal subunit	
Q9NX20	*rpl16*	O15235	*rps12*
Q9P0J6		P82664	*rps10*
P09001		P82912	*rps11*
Q16540		P82921	

Q7Z7H8	*rpl10*	P82933	
Q96A35		Q9Y2R5	
Q9BRJ2		Q9Y399	*rps2*
Q9BYD1		O60783	*rps14*
Q9BYD6	*rpl1*	P82675	
Q9NRX2		P82914	
Q9NZE8		P82932	
Q9P0M9	*rpl27*	Q96EL2	*rps3*
P49406	*rpl19*	Q9Y2R9	*rps7*
P52815		Q9Y3D3	

a) The 20 proteins of the oxidative phosphorylation pathway are separated by the different protein complexes (I-V). To 14 of the 20 proteins, a mitochondrial gene name could be assigned. Except of the proteins of Complex V, the gene names could assigned unambiguously to the human proteins. b) Out of the 30 ribosomal mitochondrial proteins of the large subunit, 12 mitochondrial gene names could be assigned. For half of the 14 proteins of the small ribosomal mitochondrial subunit the mitochondrial gene names were identified.
