# Supplementary material for: Evolution of spliceosomal introns following endosymbiotic gene transfer
Source: BMC Evol Biol. 2010 Feb 23;10:57. doi: 10.1186/1471-2148-10-57 (PMC2834692; doi:10.1186/1471-2148-10-57)
Supplement: Additional file 2 — Database sources of the complete genome and protein sequences. [file 1471-2148-10-57-S2.PDF]

Additional file 2: Database sources of the complete genome and protein sequences.

Organism	Genome sequences	Protein sequences
Arabidopsis thaliana	NCBI	NCBI
Chlamydomonas reinhardtii	JGI	JGI
Oryza sativa	EBI (Integr8)	NCBI (RefSeq)
Thalassiosira pseudonana	JGI	JGI
Plasmodium falciparum	NCBI	NCBI
Leishmania major	Sanger Institute	Sanger Institute
Dictyostelium discoideum	EBI (Integr8)	NCBI (RefSeq)
Aspergillus fumigatus	NCBI	NCBI
Candida glabrata	NCBI	NCBI
Saccharomyces cerevisiae	NCBI	NCBI
Schizosaccharomyces pombe	NCBI	NCBI
Yarrowia lipolytica	NCBI	NCBI
Danio rerio	NCBI	NCBI
Drosophila melanogaster	NCBI	NCBI
Caenorhabditis elegans	NCBI	NCBI
Homo sapiens	NCBI	NCBI
Mus musculus	NCBI	NCBI
Rattus norvegicus	NCBI	NCBI

NCBI – http://www.ncbi.nlm.nih.gov (03/2007)
JGI – http://www.jgi.doe.gov (03/2007)
EBI – http://www.ebi.ac.uk (03/2007)
