# Supplementary material for: Evolution of spliceosomal introns following endosymbiotic gene transfer
Source: BMC Evol Biol. 2010 Feb 23;10:57. doi: 10.1186/1471-2148-10-57 (PMC2834692; doi:10.1186/1471-2148-10-57)
Supplement: Additional file 3 — Percentage of codon usage for the amino acid glutamine in a) the mitochondrial genomes and in b) the nuclear genomes of the species that are included in the analysis of the parallel intron gain in the gene nad7. [file 1471-2148-10-57-S3.PDF]

**Additional file 3**: Percentage of codon usage for the amino acid glutamine in a) the mitochondrial genomes and in b) the nuclear genomes of the species that are included in the analysis of the parallel intron gain in the gene *nad7*. For each organism, the percentage of used codons CAA and CAG are listed that both code for glutamine. The average codon usage is computed for all species in each list. Percentages are taken from the codon usage database [http://www.kazusa.or.jp/codon/] (Nakamura et al. 2000).

a)

| Organism | Glutamine, CAA [%] | Glutamine, CAG [%] |
|---|---|---|
| *Dictyostelium discoideum* | 89.3 | 10.7 |
| *Thalassiosira pseudonana* | 82.6 | 17.4 |
| *Arabidopsis thaliana* | 63.1 | 36.9 |
| *Oryza sativa* | 70.4 | 29.6 |
| *Pseudendoclonium akinetukm* | 72.4 | 27.6 |
| *Ostreococcus taurus* | 81.9 | 18.1 |
| *Physcomitrella patens* | 81.4 | 16.6 |
| **Average codon usage** | **74.8** | **25.2** |

b)

| Organism | Glutamine, CAA [%] | Glutamine, CAG [%] |
|---|---|---|
| *Chlamydomonas reinhardtii* | 10.4 | 89.6 |
| *Volvox carteri* | 26.7 | 73.3 |
| *Caenorhabditis elegans* | 65.6 | 34.4 |
| *Drosophila melanogaster* | 30.2 | 69.8 |
| *Danio rerio* | 26.0 | 74.0 |
| *Homo sapiens* | 26.5 | 73.5 |
| *Rattus norvegicus* | 24.7 | 75.3 |
| *Mus musculus* | 26.0 | 74.0 |
| *Aspergillus fumigatus* | 35.8 | 64.2 |
| *Yarrowia lipolytica* | 19.0 | 81.0 |
| **Average codon usage** | **30.4** | **69.6** |
