# Supplementary material for: Evolution of spliceosomal introns following endosymbiotic gene transfer
Source: BMC Evol Biol. 2010 Feb 23;10:57. doi: 10.1186/1471-2148-10-57 (PMC2834692; doi:10.1186/1471-2148-10-57)
Supplement: Additional file 4 — Part of the multiple protein alignment of the gene nad7. [file 1471-2148-10-57-S4.PDF]

Pseudendoclonium akinetum	RGEQV	LSTDT	HIGLL	HRGTE	KLIEY	KT**VLQ**	**AL**PYF	DRLDY
Volvox carteri	NGEII	TRA**D**P	HIGLL	HRGTE	KLIEY	KT**YLQ**	**AL**PYF	DRLDY
Chlamydomonas reinhardtii	QGEII	MRA**D**P	HIGLL	HRGTE	KLLEY	KT**YLQ**	**GL**PYF	DRLDY
Ostreococcus tauri	NGEVV	ERADP	HIGLL	HRGTE	KLIEY	KN**YIQ**	**AL**PYF	DRLDY
Physcomitrella patens	NGEVV	ERAEP	HIGLL	**H**RGTE	KLIEY	KT**YLQ**	**AL**PYF	DRL**D**Y
Arabidopsis thaliana	NGEVV	ERAEP	HIGLL	**H**RGTE	KLIEY	KT**YLQ**	**AL**PYF	DRS**D**Y
Oryza sativa	NGEVV	ERAEP	HIGSL	**H**RGTE	KLIEY	KT**YLQ**	**AL**PYF	DRS**D**Y
Dictyostelium discoideum	ESENV	VRVEP	HIGLL	HRGTE	KLIEG	KT**YTQ**	**AL**PYF	DRLDY
Thalassiosira pseudonana	NGEIV	NRADP	HIGLL	HRGTE	KLIEY	KN**YVQ**	**AL**PYF	DRLDY
Yarrowia lipolytica	SGEEI	IRSDP	HVGLL	HRGTE	KLIEY	KT**YMQ**	**AL**PYF	DRLDY
Aspergillus fumigatus	NGEEI	VRADP	HVGLL	HRGTE	KLIEY	KT**YMQ**	**AL**PYF	DRLDY
Drosophila melanogaster (1)	DNETV	LNADP	HIGLL	HRGTE	KLIEY	KT**YTQ**	**AL**PYF	DRLDY
Drosophila melanogaster (2)	DGE**T**V	MRADP	HIGLL	HRGTE	KLIEY	KT**YTQ**	**AL**PYF	DRLDY
Caenorhabditis elegans (1)	EGEVI	IKAIP	HIGLL	HRATE	KLIEH	KT**YTQ**	**AL**PYF	DRLDY
Caenorhabditis elegans (2)	EGEVI	IKAIP	HIGLL	HRATE	KLIEH	KT**YTQ**	**AL**PYF	DRLDY
Homo sapiens	SGEMV	RKCDP	HIGLL	HRGTE	KLIEY	KT**YLQ**	**AL**PYF	DRLDY
Rattus norvegicus	SGEMV	RKCDP	HIGLL	HRGTE	KLIEY	KT**YLQ**	**AL**PYF	DRLDY
Mus musculus	SGEMV	RKCDP	HIGLL	HRGTE	KLIEY	KT**YLQ**	**AL**PYF	DRLDY
Danio rerio	-----	-----	-LSTL	HRGTE	KLIEY	KT**YLQ**	**AL**PYF	DRLDY

Additional file 4: Part of the multiple protein alignment of the gene *nad7*. Mitochondrial encoded sequences are shown in blue, nuclear encoded sequences are shown in black. Spliceosomal introns are marked in red, group II introns are marked in pink. The bold black amino acids surrounding the shared intron position between *Chlamydomonas reinhardtii* and the animals except of *Drosophila melanogaster* are those which are shown at the nucleotide level in Figure 4.
